# Whole-Genome Sequencing of *Streptomyces* sp. Strain UYFA156, a Cultivar-Specific Plant Growth-Promoting Endophyte of *Festuca arundinacea*

**DOI:** 10.1128/MRA.00722-19

**Published:** 2019-09-19

**Authors:** Patricia Vaz Jauri, Martín Beracochea, Belén Fernández, Federico Battistoni

**Affiliations:** a Departamento de Bioquímica y Genómica Microbianas, Instituto de Investigaciones Biológicas Clemente Estable, Montevideo, Uruguay; Indiana University, Bloomington

## Abstract

*Streptomyces* spp. produce many and diverse bioactive metabolites. Plant growth-promoting (PGP) activity by *Streptomyces* spp. has been reported repeatedly; however, the mechanisms are largely unknown. We report the sequencing of the genome of a PGP endophytic *Streptomyces* sp. strain, which will contribute to the understanding of the underlying mechanisms for growth promotion.

## ANNOUNCEMENT

Endophytic bacteria have been increasingly seen as relevant partners influencing plant health (1, 2). *Streptomyces* sp. strain UYFA156 was isolated from seeds of Festuca arundinacea cv. SFRO Don Tomás (tall fescue). Although bacteria of the genus *Streptomyces* are studied mostly for their notable antibiotic production ability (3), this strain stands out not for its biocontrol activity but for another plant health-associated trait. The strain UYFA156 has plant growth-promoting (PGP) activity in its host, F. arundinacea cv. SFRO Don Tomás, but not in another tall fescue cultivar, F. arundinacea cv. Tacuabé (4). Several phenotypes associated with PGP activity (5) in bacteria have been searched for in UYFA156, such as indole-acetic acid production, K and P solubilization, the presence of the gene *nifH*, and nitrogenase activity, through the acetylene reduction assay, and none gave positive results (4). In addition, its true endophytic nature has been shown (P. Vaz Jauri, C. Taulé, M. C. de los Santos, B. Fernández, A. Di Paolo, J. R. Sotelo-Silveira, and F. J. Battistoni, submitted for publication) in both the compatible interaction with F. arundinacea cv. SFRO Don Tomás and the incompatible interaction with F. arundinacea cv. Tacuabé. We aim to uncover the molecular basis of this beneficial interaction, and thus we sequenced the bacterial genome, which will be used as a reference for future work.

UYFA156 was isolated from surface-sterilized F. arundinacea cv. SFRO Don Tomás seeds. Briefly, 1 g of seeds was macerated with a mortar and pestle under sterile conditions, and dilutions of the resulting suspension were planted onto tryptic soy agar (TSA) medium and incubated at 30°C as described previously (5). UYFA156 was further purified under the same conditions and then kept as spores in 25% glycerol at –80°C. For genomic DNA extraction, UYFA156 was cultivated in 5 ml of tryptic soy broth (TSB) in 50-ml tubes at 30°C and 180 rpm for 24 h. DNA was extracted using the cetyltrimethylammonium bromide (CTAB) method (Joint Genome Institute protocols [http://1ofdmq2n8tc36m6i46scovo2e-wpengine.netdna-ssl.com/wp-content/uploads/2014/02/JGI-Bacterial-DNA-isolation-CTAB-Protocol-2012.pdf]). Extracted DNA was sequenced by Macrogen, Inc. (South Korea), using PacBio RS II technology (Pacific Biosciences) and single-molecule real-time (SMRT) cell 8Pac v.3, the DNA polymerase binding kit P6, and DNA sequencing reagent 4.0 v.2 with 185× coverage (*n* = 1,316,422,473 subreads) and a subread *N*_50_ value of 16,014 bp.

The raw PacBio reads were corrected, trimmed, and *de novo* assembled using Canu v.1.8 (genomeSize, 15 m). The result of the assembly was two contigs with an *N*_50_ value of 6,823,997 bp. Based on the assembly information, we report that the UYFA156 genome consists of 7,131,381 bp divided into one chromosome of 6,823,997 bp and a megaplasmid of 307,384 bp. Like all *Streptomyces* species, UYFA156 has a high GC content (73.4%). Seven copies of rRNA operons were found in UYFA156, which is consistent with the ability of this strain to grow rapidly in highly complex media (6) such as the inside of plants. Using FastANI v.1.1 (7), the most similar genome was found to belong to the strain Streptomyces albidoflavus SM254, with an average nucleotide identity value of 96.27% (8), which was studied for being a potent pathogen antagonist and was reported to have similar genomic characteristics.

Similar to strain *S. albidoflavus* SM245, UYFA156 has several secondary metabolite gene clusters, as revealed by antiSMASH v.5.0.0 (9), including nonribosomal peptide synthetases (NRPS), polyketide synthases (pKS), ectoine, terpenes, and desferroxamine, among others.

### Data availability.

The sequence data of *S. albidoflavus* UYFA156 have been deposited in DDBJ/ENA/GenBank under the BioSample number SAMN11663544, BioProject number PRJNA543336, and genome accession numbers CP040466 and CP040467 for the chromosome and the megaplasmid, respectively. The raw sequence data can be accessed through the NCBI Sequence Read Archive (SRA) under the identifier SRX5891284.
